# Role of preoperative endoscopic clipping in laparoscopic distal gastrectomy for early gastric cancer

**DOI:** 10.1097/MD.0000000000013165

**Published:** 2018-11-09

**Authors:** Dae Hwa Park, Hee Seok Moon, Ji Young Sul, In Sun Kwon, Gee Young Yun, Seo Hee Lee, Jae Ho Park, Ju Seok Kim, Sun Hyung Kang, Eaum Seok Lee, Seok Hyun Kim, Jae Kyu Sung, Byung Seok Lee, Hyun Yong Jeong

**Affiliations:** a Division of Gastroenterology, Department of Internal Medicine, Daejeon Veterans Hospital; b Division of Gastroenterology, Department of Internal Medicine; c Department of General Surgery; d Clinical Trials Center, Chungnam National University Hospital, Daejeon, Republic of Korea.

**Keywords:** additional resection, early gastric cancer, laparoscopic distal gastrectomy, preoperative endoscopic clipping, resection margin

## Abstract

In this study, we evaluate the usefulness of preoperative endoscopic clipping for early gastric cancer (EGC) localization in laparoscopic distal gastrectomy.

We retrospectively screened all consecutive patients who underwent laparoscopic distal gastrectomy for EGC by 1 surgeon at Chungnam National University Hospital between January 2014 and December 2016. Patients who underwent combined surgery and patients who had tumors at the lower third of the stomach were excluded. Endoscopic clipping was performed prior to surgery by specialized endoscopists. During the operation, endoscopic metal clips were found using surgical devices, and laparoscopic vessel clips were attached on the presumed site; thereafter, intraoperative radiographs were obtained for confirmation.

We analyzed a total of 196 patients; of them, 101 were classified into the clipping group (CG) and 95 into the non clipping group (NCG). The 2 groups were comparable regarding their demographic characteristics. The CG showed less additional resection (2 of 101 patients [2.0%] vs 9 of 95 patients [9.4%], *P* = .021) and better outcomes in terms of the operation time (*P* = .000), duration of hospital stay (*P* = .036), and postoperative atelectasis (*P* = .001) than the NCG.

Preoperative endoscopic clipping was helpful in determining the exact resection margin in laparoscopic distal gastrectomy for EGC.

## 1 Introduction

In Korea, gastric cancer is the first most common in men and the fourth most common cancer in women,^[1]^ also it is the second leading cause of cancer-mediated death.^[2]^ According to the nationwide survey of 69 hospital, the proportion of early gastric cancer has increased, reaching 61% in 2014.^[1]^ Recently, the increased use of endoscopy and greater reach of surveillance programs have enabled earlier diagnosis.^[3]^

Laparoscopy-assisted distal gastrectomy was first reported in 1994.^[4]^ Owing to a fast recovery after surgery and better quality of life, laparoscopic gastrectomy has become a widely used treatment for early gastric cancer (EGC), especially in Korea and Japan.^[5–7]^

However, it is difficult to identify small lesions in EGC during laparoscopic surgery even for a skilled surgeon.^[8]^ Because EGCs do not involve the serosal layer, the location of a tumor cannot be determined by the laparoscopic view alone.^[2]^ Without precise preoperative localization, a large portion of the stomach has to be dissected to ensure complete eradication of the cancer tissue. Conversely, some patients still need subsequent surgical resections owing to positive resection margins.^[8]^ Thus, correct identification of the tumor location is very important, especially for gastric cancer in the middle third of the stomach.^[7]^

Various methods have been introduced to locate the tumor: preoperative endoscopic clipping, intraoperative endoscopy, intraoperative ultrasonography, intraoperative portable radiography, and intraoperative tattooing.^[9]^ However, to our knowledge, no studies have demonstrated how effective these methods in laparoscopic gastrectomy actually are in comparison with a non-marking group. In our institution, preoperative endoscopic clipping was performed to mark the location of tumors; in this study, we aimed to analyze how effective this method is.

## 2 Materials and methods

### 2.1 Study population and design

We retrospectively reviewed the medical records of 247 patients who underwent laparoscopic distal gastrectomy by 1 surgeon (JYS) at Chungnam National University Hospital (CNUH) between January 2014 and December 2016. The surgeon had more than 10 years of laparoscopic gastrectomy experience. Laparoscopic distal gastrectomy was indicated for patients with cT1–2N0M0 tumors based on the preoperative assessment in our institution.

During the study period, preoperative endoscopic clipping was performed only in some patients in the early part of the study; however, clipping was gradually applied to more patients over time because it was thought to be effective in marking tumor locations before surgery. In other words, more patients were included in the non clipping group (NCG) in the early part and in the clipping group (CG) in the latter part. This study was approved by the Chungnam National University Hospital Institutional Review Board (IRB file No. CNUH 2018–01–007).

Gastric resection and anastomosis construction were performed extracorporeally in all cases; the reconstruction methods were selected depending on the location of the tumor and the size of the remnant stomach. Further, D1 + B or D2 lymphadenectomy was performed in accordance with the Japanese Gastric Cancer Treatment Guideline (4th English edition).^[10]^

**Figure 1:**
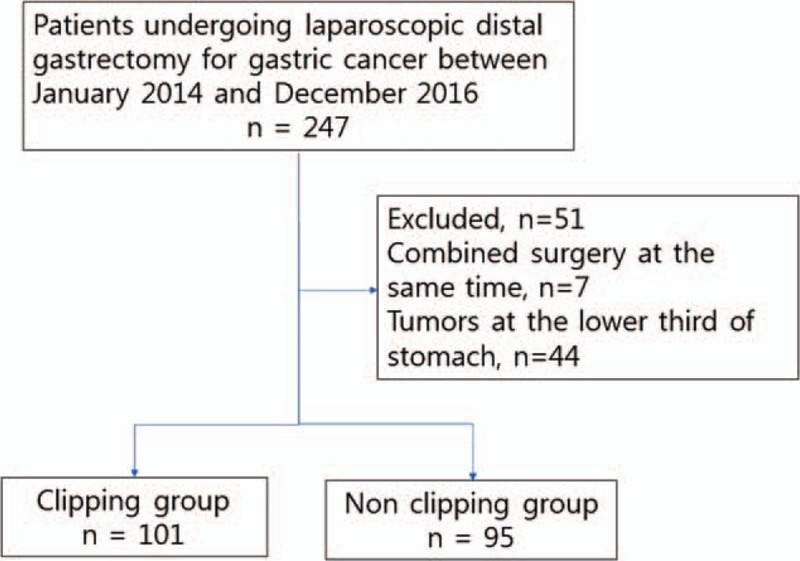
Flow chart showing the inclusion of the patients in the study.

Of the 247 enrolled patients, 7 patients and 44 patients were excluded because they underwent combined surgery at the same time and because they had tumors at the lower third of their stomach, respectively. Finally, we analyzed and classified 101 patients into the CG and 95 patients into the NCG (Fig. 1).

### 2.2 Preoperative clipping procedure

**Figure 2:**
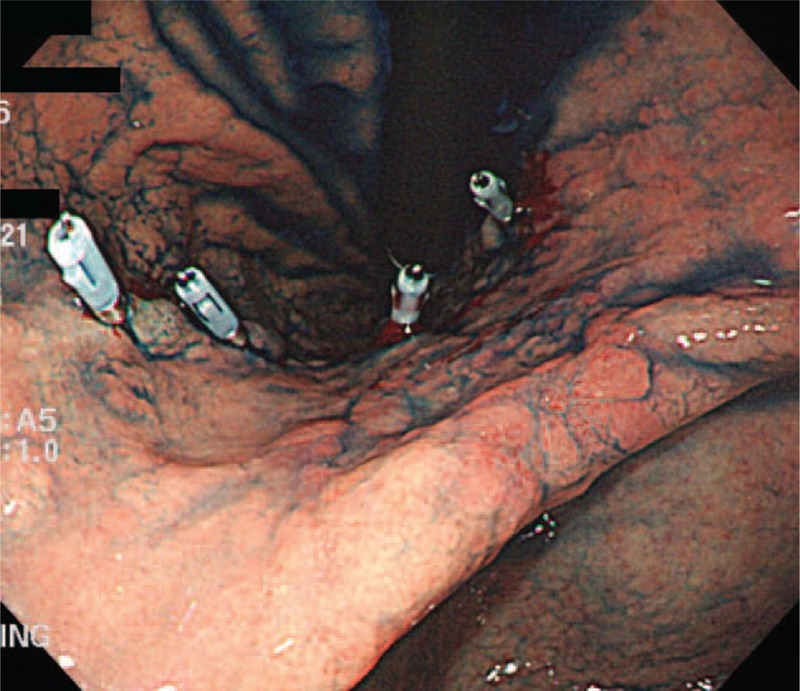
Preoperative localization procedures. After application of the indigo carmine dye to identify the lesion boundary clearly, several metallic clips were applied 2 or 5 cm proximal to the upper border of the tumor. At this time, one or more clips were mounted on the anterior wall.

All patients underwent preoperative endoscopic clipping in the early morning on the day of surgery or 1 day before surgery. The location of the clipping was determined on the basis of their previous endoscopic biopsy results. When the degree of differentiation was well or moderate, clipping was performed 2 cm more proximally than the upper border of the tumor; when the degree of differentiation was poor, clipping was performed at 5 cm proximally. Clipping was performed by two specialized endoscopists with an endoscopic experience of more than 10 years, and no other marking methods other than metal clipping, such as dye injection, were used (Fig. 2).

### 2.3 Intraoperative localization method

**Figure 3:**
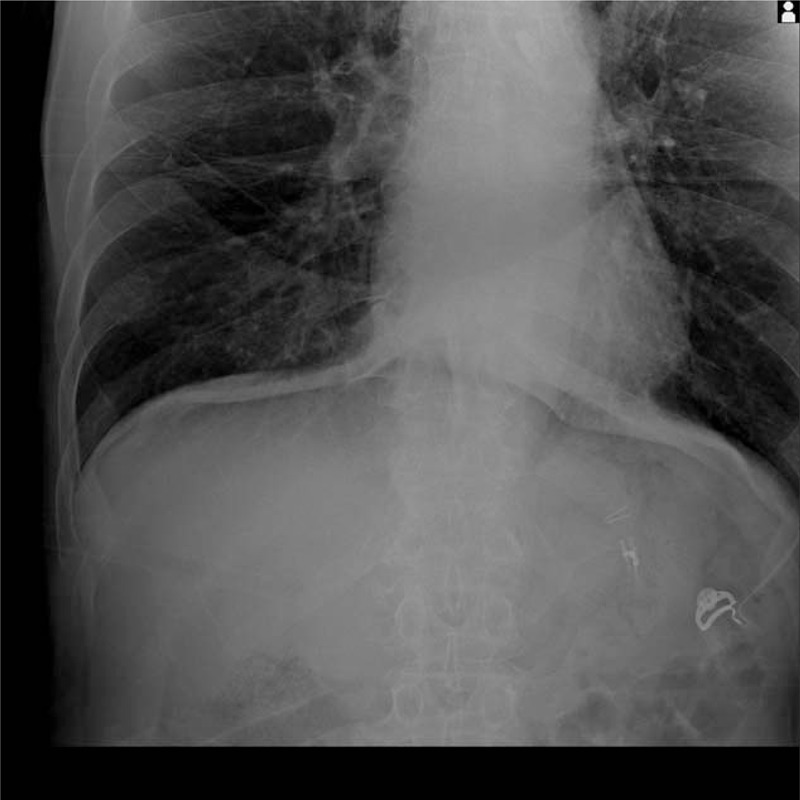
Intraoperative radiography showing the location of the metallic clips and endoscopic clips.

**Figure 4:**
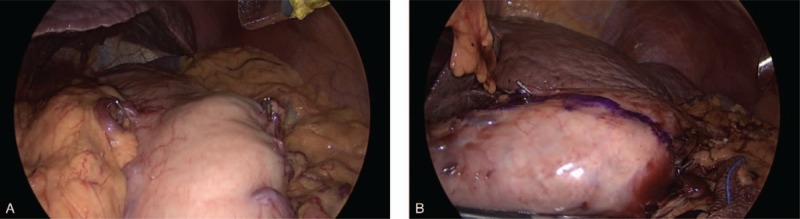
Intraoperative localization procedure. Two metallic clips were applied to the external surface of the stomach (A), Thereafter, the transection line was drawn using gentian violet based on the intraabdominal radiography (B).

**Figure 5:**
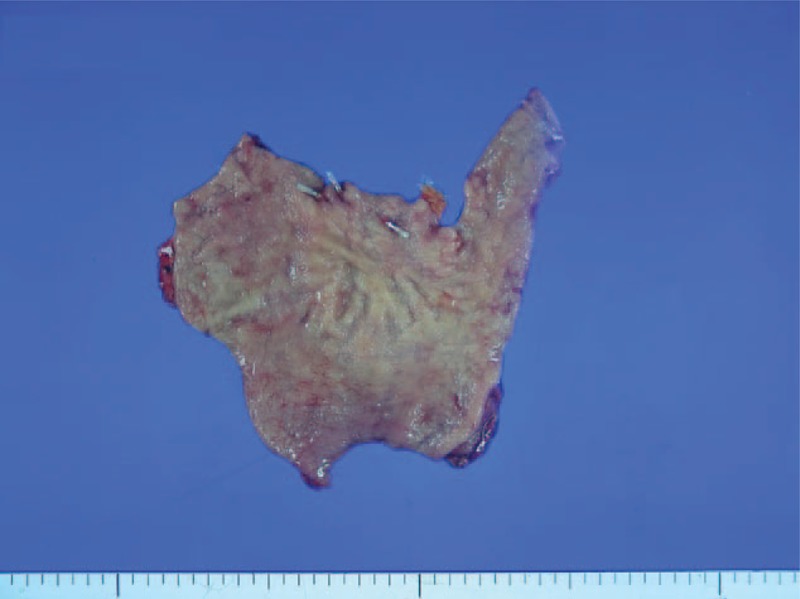
Resected specimen of distal gastrectomy. The resection line was determined adjacent to the endoscopic metal clips applied before surgery.

Because the location of the endoscopic clip (HX-610–090L, Olympus, Tokyo, Japan) was not visible through the laparoscope, the surgeon used a surgical device to sweep the stomach wall. At this time, we could find the position of the clip with the sound and feeling of touch within a few minutes and attached a metallic vessel clip (176630, Covidien, USA) to the peritoneal side of the gastric wall, which was presumed to be the position of the endoscopic metallic clip. After the laparoscopic clips were applied, intraoperative radiographies were obtained to confirm the location of the clips (Fig. 3). The proximal resection line was then determined in accordance with the correlation between the endoscopic clips and laparoscopic clips. Thereafter, the surgeon drew a resection line on the serosal surface using gentian violet (pyoktanin blue solution) based on the intraoperative radiography (Fig. 4). After resecting the stomach, the surgeon moved the resected specimen out of the body and confirmed the grossly negative margins by opening the specimen and consequently identifying the metallic clips (Fig. 5). Frozen sectioning was not performed during the surgery when the gross margin seemed sufficient. When sufficient proximal margins were not obtained or tumor cells were present in the frozen section, additional resection was performed

### 2.4 Statistical analysis

IBM SPSS ver. 18.0 (IBM Co., Armonk, NY) was used for the statistical analyses. The *χ*^*2*^ test and student *t*-test were used for the comparisons between the CG and NCG, as appropriate. A *P* value of < .05 was considered statistically significant.

## 3 Results

Of the 196 patients enrolled in this study, 101 (51.5%) were classified into the CG and 95 (48.5%) into the NCG. Preoperative clipping was performed without complications in all patients, and all applied endoscopic and laparoscopic metallic clips were easily detected by intraoperative abdominal radiography as radio-opaque materials.

Both groups were comparable regarding their demographic characteristics, that is, age, sex, body mass index, smoking history, abdominal surgery history, American Society of Anesthesiologists (ASA) score, reconstruction method, location of tumor. However, in the early part of the study period, the NCG was more and the CG was more in the latter part (Table 1).

**Table 1:**
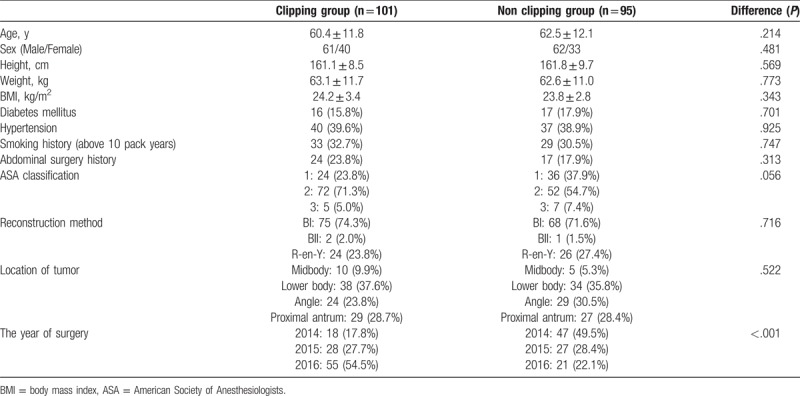
Patient demographics.

The clinicopathological outcomes are shown in Table 2. There were also no significant differences in the size of tumor (*P* = .798), T and N stage (*P* = .154 and *P* = .667, respectively), differentiation of tumor (*P* = .096), albumin, and hemoglobin level decrease on the day of surgery (*P* = .181 and *P* = .053, respectively), mean time to first liquid diet (*P* = .259), and complications requiring reoperation (*P* = .905) between the 2 groups. However, the CG showed better results in terms of the operation time (*P* < .001), duration of hospital stay (*P* = .036), and postoperative atelectasis (*P* = .001) than the NCG. Additional resection was performed in 11 patients (5.6%): 2 (2.0%) in the CG and nine (9.4%) in the NCG. At the same time, the length of the proximal margin was not statistically significant in both groups, but was slightly shorter in the CG (*P* = .138) than in the NCG. Since clipping was done more in the latter part of the study period, we conducted a yearly analysis of meaningful data to exclude the bias. As a result, although the sample size was insufficient and some items showed no statistically significant *P* value by year, the result were overall similar to those of whole period analysis. In other words, in the yearly analysis, the CG also showed better results of operation time, hospital stay and postoperative atelectasis compared to the NCG (Table 3).

**Table 2:**
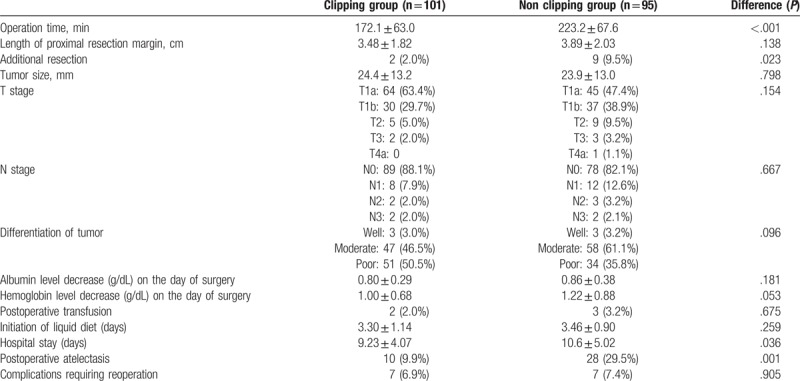
Clinicopathological outcomes of all study period (clipping group vs non clipping group).

**Table 3:**
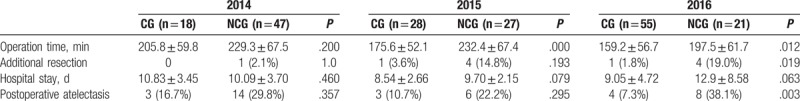
Yearly analysis of major clinical results.

The clinicopathological outcomes of these 11 patients are summarized in Table 4. Two patients had tumors on the resection edges, and three patients had tumor cells in the frozen section. The remaining 5 patients underwent additional resection because the resection margins were too close to the tumor when the initial transection specimen was identified by the surgeon. In all patients, the pathologic results confirmed negative tumor cells at the final resection margin.

**Table 4:**
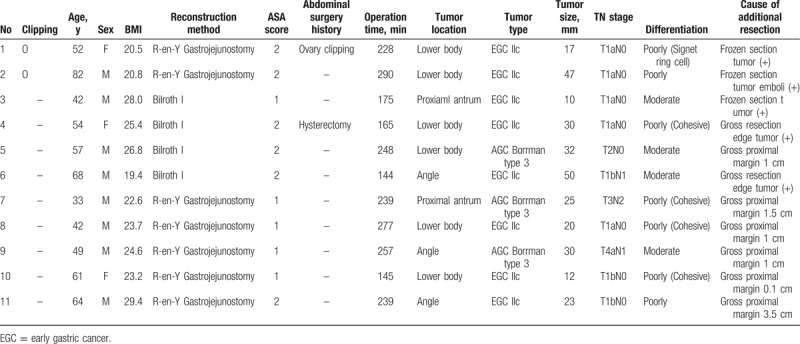
Clinicopathologic results of the 11 additional resection patients (two in the clipping group and nine in the non clipping group).

## 4 Discussion

Various methods of intraoperative tumor localization have been studied because EGC lesions cannot be identified by inspecting the serosal surface, and the ability to identify lesions by palpating or opening the stomach is not available in laparoscopic distal gastrectomy.^[11]^ Accurate localization of the tumor prior to or during gastrectomy and determination of the appropriate resection line would help avoid total gastrectomy and determine the appropriate type of reconstruction after subtotal gastrectomy.^[12,13]^ In addition, accurate localization increases the likelihood of complete resection and minimizes additional resection in the surgical field.^[13]^ Microscopic diseases at the resection line affect patient long-term survival; thus, surgeons should ensure that resection lines are tumor free.^[7,14,15]^ Thus, if a sufficient proximal resection margin can be achieved, laparoscopic distal gastrectomy can be a curative treatment option for EGCs in the middle third of the stomach. ^[7,16]^

To date, several techniques for intraoperative identification of tumors have been reported,^[17]^ such as preoperative gastroscopy with indigo carmine dye injection,^[7]^ autologous blood tattooing,^[11]^ intraoperative endoscopy,^[2,18,19]^ intraoperative radiography,^[9,13]^ intraoperative ultrasonography,^[2,17,20]^ and even endoscopic clipping with CT gastrography.^[21]^ However, each method has several disadvantages. Intraoperative endoscopy may disturb the surgical field because it distends the upper gastrointestinal tract. Furthermore, intraoperative endoscopy, ultrasonography, and endoscopic clipping with CT gastrography are costly and require special instruments, special training, and additional labor.^[22]^ Intraoperative radiography requires waiting for the radiographer to arrive, which prolongs the time to obtain an intraoperative image, and the 2-dimensional view of intraoperative radiography could provide an incorrect information on the location of a tumor.^[2]^ Endoscopic dye injection can diffuse into the serosal surface and eventually obscure the precise tumor location.^[19]^ Because of these disadvantages, it is not yet clear whether the marking of the tumor location should be indicated in laparoscopic distal gastrectomy. A previous study performed tattooing using India ink in laparoscopic colorectal cancer surgery and showed shorter operation time, correct resection, and less blood loss compared with their non-tattooing group.^[23]^ However, to our knowledge, this is the first study to compare 2 groups with and without marking of the tumor location in laparoscopic gastric cancer surgery.

In this study, the CG showed a lower incidence of additional resection and better outcomes in terms of the operation time, hospital stay duration, and postoperative atelectasis than the NCG. It is possible that the better results in hospital stay and postoperative atelectasis in the CG were due to shorter operation time. However, there was no statistically significant difference in the postoperative reduction of the hemoglobin and albumin levels and complications requiring reoperation.

Although most studies have reported that an additional resection was rarely needed when resection was performed after tumor location marking, Kim et al^[9]^ reported that 2 of their 29 patients (6.9%) required additional resections using intraoperative radiography with radio-opaque markers after preoperative endoscopic clipping. Moreover, Kawakatsu et al. reported that 6 of their 556 patients (1.1%) required additional resections using intraoperative endoscopy after preoperative endoscopic clipping.^[19]^

The most important purpose of our lesion marking using endoscopic clipping was to obtain an adequate resection margin while avoiding excessive resection. In this study, a fewer additional resections were required in the CG, and although there was no statistically significant difference, the proximal resection margin tended to be shorter. Thus, clipping seems to have helped determine the appropriate resection margin while avoiding excessive resection. In our institution, endoscopic clipping was performed before operation, and the stomach wall was swept using surgical devices to find the clips in the laparoscopic view. However, other studies have reported other methods, such as intraoperative endoscopy and intraoperative ultrasonography. Since we used only a single method, further studies are needed to confirm which method is more accurate and superior.

This study has some limitations. First, this study was a retrospective study, in which the results were obtained by a single surgeon in a single center and the sample size was small. Second, this study was not a randomized controlled trial, and preoperative endoscopic clipping was considered to be effective and was applied to an increasing number of patients during the study period. As a result, the CG included more patients with more recent surgery than the NCG. Therefore, this may have affected the outcomes of the surgeon's experience, such as the operation time and duration of hospital stay. To overcome this bias, we conducted a yearly analysis of the major results. Third, the latest surgical procedure is total laparoscopic surgery with intracorporeal anastomosis. However, laparoscopy-assisted gastrectomy with extracorporeal anastomosis was performed in this study. Fourth, laparoscopic distal gastrectomy was performed on patients suspected of having EGCs based on preoperative gastroscopy and CT scan, but unfortunately the staging of this method is not completely accurate, and some of the patients included in this study were diagnosed with advanced gastric cancer in postoperative histopathology. Finally, the distance between the clips and the tumor and the distance between the endoscopic clips and laparoscopic vessel clips were unavailable in this study.

In conclusion, preoperative endoscopic clipping in EGC was helpful in determining the exact resection margin. In this case, surgeons may depend on the location of the endoscopic clips in determining the resection line; therefore, adequate communication between the surgeon and the endoscopist is essential.

## Author contributions

**Conceptualization:** Hee S. Moon.

**Data curation:** Dae H. Park, Ji Y. Sul, Gee Y. Yun, Seo H. Lee, Jae H. Park.

**Formal analysis:** Dae H. Park.

**Investigation:** Ji Y. Sul, Ju S. Kim, Sun H. Kang, Eaum S. Lee, Seok H. Kim.

**Methodology:** Hee S. Moon, In S. Kwon.

**Supervision:** In S. Kwon, Jae K. Sung, Byung S. Lee, Hyun Y. Jeong.

**Writing – original draft:** Dae H. Park.

**Writing – review & editing:** Hee S. Moon.
